# Cannabis use and perceptions among rural and urban cancer patients and survivors from a Pennsylvania cancer center

**DOI:** 10.1007/s00520-026-11061-7

**Published:** 2026-10-09

**Authors:** Jeremy Ireland, Leah Holben, Cheryl L. Thompson, Junjia Zhu, Hyma V. Polimera, Cristina I. Truica

**Affiliations:** 1 Department of Public Health Sciences, Pennsylvania State University College of Medicine, 500 University Drive, Hershey, PA 17033 USA; 2 Penn State Cancer Institute, 500 University Drive, Hershey, PA CH6917033 USA; 3 Department of Hematology/Oncology, Fox Chase Cancer Center, 333 Cottman Ave, Philadelphia, PA 19111 USA; 4 Department of Medicine, Division of Hematology/Oncology, Penn State Cancer Institute, 500 University Drive, Hershey, PA 17033 USA

**Keywords:** Cancer, Cannabis, Marijuana, Attitudes, Perceptions, Rural, Urban

## Abstract

**Purpose:**

Cannabis use has become more common among cancer patients for symptom management. Given the underrepresentation of rural patients in prior research, our study explored cannabis use and perceptions among cancer patients, with a focus on comparing rural and urban/suburban patients.

**Methods:**

Adult cancer patients at the Penn State Cancer Institute completed an anonymous survey on cannabis use and attitudes. Eligible participants had a cancer diagnosis and had been in active treatment within the previous year. We compared rural and urban/suburban patients using Fisher’s exact test. The results were further examined using a multivariable logistic regression model, controlling for area of residence and sociodemographic factors.

**Results:**

Among the 186 patients surveyed, 26.1% had used cannabis since diagnosis. Rates were not significantly different between rural and urban/suburban patients, and area of residence was not associated with cannabis use in multivariable regression analysis. Symptom improvement was reported for insomnia (89.7% of users), appetite (85.7%), mood (83.7%), joint pains/stiffness (83.3%), stress/anxiety/depression (81.6%), cancer-related pain (80.7%) and digestive symptoms (67.9%), with no differences by area of residence. Urban/suburban patients were more likely to believe that cannabis helps with nausea/vomiting (*p* = 0.019), more likely not to use cannabis due to concerns about interactions with medications (*p* = 0.002), and more likely to obtain cannabis from dispensaries (*p* = 0.046).

**Conclusion:**

Cannabis use was common in our study, with similar rates of use and attitudes towards cannabis between rural and urban/suburban patients. A majority of users reported improvement in most cancer-related symptoms assessed, highlighting its potential role in cancer symptom management.

## Introduction

Interest in the medical use of cannabis and cannabinoids has grown, particularly for alleviating symptoms such as sleep disturbance, pain, and anxiety in cancer patients [1–4].

In the U.S., cannabis is allowed or regulated for medical use in 40 states and adult nonmedical use in 24 states [5]. Recently, certain FDA-approved and state-licensed medical cannabis products were moved to Schedule III, while federal law continues to treat cannabis as Schedule I [6]. The rapidly evolving and widely varying legal landscape has created disparities and stigma around use, access to cannabis, and perceived risks [7].

Cannabis use has grown more prevalent in recent years both in the general U.S. population [8, 9] and even more so among people with a cancer history [10–12]. A population-based analysis from the US National Health and Nutrition Examination Survey reported that past-month cannabis use among adults with a cancer history aged 20–60 years rose from 5.6% to 12.2% between 2005–06 and 2013–14, compared to an increase from 6.4% to 7.2% among those without cancer [10]. A 2024 systematic review reported that cannabis use rates among cancer patients and survivors based on 6 national samples ranged between 4.8% and 22% [13], while single-site studies have reported rates over 30% [2, 14, 15], noting that estimates vary by legal environment and measurement window (e.g., ‘use since diagnosis’ vs. past 3-month use). A multi-state survey (2021–2023 data) across 12 NCI-Designated Cancer Centers with varying cannabis-access legal statuses reported 32.9% use since diagnosis [3], and a 2024 study which utilized a registry-linked, population-based sample in Washington State reported 41.3% post-diagnosis use among recently diagnosed patients [4].

Cannabis use by cancer patients or survivors is positively correlated with prior cannabis use [2, 16], younger age [1, 2, 4, 7, 11, 17–19], male sex [1, 18, 20], African American race [1, 18, 20], depression [1, 20], heavy alcohol use [1, 17, 20], smoking (current or former) [1, 7, 11, 17, 20], lower education levels [4, 19], pain [12], late-stage cancer [4, 16], and receiving active treatment [21]. In addition, higher symptom burden among cancer patients is linked to concurrent substance use among cannabis users [22].

While observational studies frequently report symptom improvement among cannabis users [2, 3, 10, 13, 23], evidence for benefits in cancer-related symptoms remains limited [24, 25]. A 2025 systematic review and meta-analysis of 98 studies reported reductions in pain and anxiety in pre–post analyses, more limited evidence for appetite, chemotherapy-induced nausea and vomiting (CINV), and insomnia, no effects on several other symptoms or overall QoL, and increased adverse events with some cannabinoid products [26]. A 2023 umbrella review of 101 meta-analyses found moderate-certainty evidence in cancer populations that cannabinoids may improve sleep disturbance but also increase risks of serious adverse events, nervous system adverse events, gastrointestinal disorders or nausea, and poorer tolerability; GI effects may vary by cannabinoid composition, dose, formulation, and clinical context [27, 28]. The 2024 American Society for Clinical Oncology (ASCO) guideline offered only a weak recommendation to consider cannabis-based medicines as adjuncts for refractory CINV, with insufficient evidence for other cancer-related symptoms or treatment-related toxicities [25]. Frequent and non-medical use has also been associated with poorer mental health–related quality of life and depression in survivor samples [29, 30], a concern given that daily use is common in some oncology populations; for example, 35% of current cannabis users in a survey of women with gynecologic malignancies in California and Colorado reported daily use [31].

Most published studies regarding cannabis use in U.S. cancer patients have been conducted in large, urban cancer centers using electronic questionnaires, leaving unanswered questions about cannabis use, perceptions, access, and sourcing outside metropolitan settings. Rural cancer patients often have long travel times and are more likely to experience limited access to medical providers, and as such are underrepresented in clinical trials, all of which affect the quality of care and health outcomes [32]. Rural counties have a higher incidence of several cancer types, including those related to tobacco use and cancers preventable by screening, and higher cancer death rates [33]. These differences may reflect higher prevalence of risk factors such as smoking, obesity, and physical inactivity; disparities in health care access, timely diagnosis, and treatment [33]; and, in some regions, environmental or occupational exposures such as agricultural pesticide-related exposures [34]. Therefore, we aimed to examine cannabis use and attitudes among cancer patients in the Penn State Cancer Institute’s (PSCI) mixed rural–suburban–urban catchment, which includes 19 Appalachian counties and a sizable rural patient base (> 12%), by comparing responses by residence.

## Methods

From June 2023 to June 2024, we conducted an anonymous, cross-sectional survey of adults with a cancer history during their in-person visits to PSCI. Recruitment occurred across multiple medical oncology clinics and waiting areas, including infusion suites. Potentially eligible patients were approached using an IRB-approved script and invited to participate in the study. After obtaining verbal consent, participants completed a paper questionnaire that did not collect personal protected information. We chose in-person administration to enhance participation among individuals from rural areas who might have limited internet access or computer literacy. Eligible participants were ≥ 18 years old, able to read and understand English, had a cancer diagnosis, and received at least part of their cancer care at PSCI within 12 months of survey completion. Family assistance with survey completion was discouraged but permitted. Patients with multiple visits during the study period were asked to complete the survey only once.

Completed surveys were returned to clinic staff and deposited in a secure collection box, retrieved daily. Survey data were managed in REDCap (Research Electronic Data Capture) hosted at Penn State College of Medicine. Paper responses were entered manually by 2 study team members with independent cross-checks and resolution of discrepancies. The survey was adapted for content and length from a survey initially developed by B Wortser et al [35], and was reviewed by providers including nurses, clerks, physician assistants, and a patient advocate, with feedback incorporated into the final wording. The survey included questions about cannabis use, reasons for/against use, and perceptions of benefits and risks. We also collected demographic information including age, race, gender, education, income, self-reported area of residence (rural/urban/suburban), cancer diagnosis, and cancer treatments. Cannabis was defined in the survey as “marijuana, cannabis concentrates, edibles, lotions, ointments, tinctures containing cannabis, any CBD-containing products, pharmaceutical or prescription cannabinoids, or other products made with cannabis.” This study and questionnaire were approved by the Penn State Institutional Review Board prior to study initiation, and all research was conducted in accordance with the Declaration of Helsinki.

Survey responses were summarized by frequencies and percentages for categorical variables and by means and standard deviations for age. All participants completed at least one non-demographic survey item, but no item was completed by all participants. Proportions used the number of respondents with non-missing data for each item. Responses by patients from rural areas were compared to those who lived in either urban or suburban areas using Fisher’s exact test for categorical variables and the Wilcoxon rank-sum test for age. We fit a multivariable binary logistic regression with *cannabis use since diagnosis*as the dependent variable. The initial model included area of residence (rural vs. urban/suburban), age, employment, race, gender, education, income, and marital status. In the final model, area of residence, the primary exposure, and core demographic variables (age, race, and gender) were retained regardless of statistical significance, whereas other non-significant covariates were removed. Ninety-five percent confidence intervals for proportions were calculated using the Wilson score method with a continuity correction. A two-sided significance level of 0.05 was used, with statistical analysis performed with R (version 4.0.2) [36].

## Results

186 patients participated in the survey, of whom 58.9% were female, 89.9% white, and the mean age was 62.1 years (SD = 14.1; range = 20–91) (Table 1). 49.5% (*n* = 92) lived in rural areas, 41.4% (*n* = 77) in suburban areas and 5.4% (*n* = 10) in urban areas (3.8% did not respond to this item). 52.3% were living comfortably on their present income, while 14.7% reported finding it difficult or very difficult to get by. 52.2% had some college education, 32.0% were employed, and 43.8% were retired. 59.4% had late-stage cancers and the most common cancer type was breast (27.8%), followed by lung (15.0%) and melanoma/skin (12.2%). 67.9% had received chemotherapy and the most common primary health care source was Medicare (46.1%). Rural patients were more likely to have Tricare and less likely to have Medicare.

**Table 1:** Sociodemographic and clinical characteristics, stratified by area of residence

		Overall (*n *= 186)		Area of Residence (*n* = 179)		
				Rural (*n* = 92)	Urban/Suburban (*n* = 87)	
		Response (*n*)	%	%	%	*p*
Age		179				0.10
	< *65 years*		52.5	59.3	46.0	
	≥ *65 years*		47.5	40.7	54.0	
Race†		179				0.14
	*White*		89.9	93.4	86.2	
	*Black*		5.6	3.3	8.0	
	*Other*		4.5	3.3	5.7	
Gender		180				0.45
	*Female*		58.9	55.4	62.1	
	*Male*		41.1	44.6	37.9	
Income		177				0.23
	*Living comfortably*		52.3	47.8	57.5	
	*Not living comfortably*		47.7	52.2	42.5	
Education		180				0.13
	*Some college*		52.2	46.2	58.1	
	*No college*		47.8	53.8	41.9	
Marital status		180				0.42
	*Married/living as married*		68.9	72.5	66.3	
	*Not married*		31.1	27.5	33.7	
Occupational status		178				0.75
	*Employed*		32.0	33.7	31.4	
	*Not employed*		68.0	66.3	68.6	
Cancer stage		133				0.86
	*Early (stages 0–2)*		40.6	39.1	41.3	
	*Late (stages 3–4)*		59.4	60.9	58.7	
Recurrent/metastatic		155	38.7	37.0	41.7	0.62
Cancer type‡		180				
	*Breast*		27.8	26.4	29.1	0.74
	*Lung*		15.0	16.3	13.8	0.68
	*Skin/melanoma*		12.2	8.7	14.9	0.25
	*Lymphoma*		11.1	8.7	13.8	0.35
	*Other*		58.7			
Treatment received‡		162				
	*Chemotherapy*		67.9	70.4	64.6	0.50
	*Radiation therapy*		47.5	43.2	51.9	0.34
	*Immunotherapy*		37.0	33.3	40.5	0.41
	*Targeted therapy*		29.6	28.4	31.6	0.73
	*Steroids*		28.4	22.2	34.2	0.11
	*Hormone therapy*		17.3	11.1	24.1	0.04*
	*Clinical trial*		10.5	11.1	10.1	1.0
	*Stem cell/bone marrow transplant*		10.5	9.9	10.1	1.0
	*Other*		11.1			
Health care source‡		178				
	*Medicare*		46.1	35.9	54.0	0.02*
	*Plan through employer/union*		43.8	45.7	41.4	0.65
	*Medicaid/state program*		12.9	14.1	11.5	0.66
	*Plan bought on own*		9.0	9.8	8.0	0.80
	*TRICARE/VA/military*		7.3	10.9	2.3	0.03*
	*Other*		1.7			

Response (*n*) indicates the number of respondents with non-missing data for each item; percentages were calculated among respondents to that item within each column. Area-specific percentages and *p* values are based on the 179 respondents who reported area of residence

†Race was analyzed as a binary variable for rural vs. urban/suburban comparisons (white patients compared to non-white patients)

‡Percentages sum to greater than 100 because more than one option could be chosen

26.1% (95% CI: 20.0, 33.2) of respondents had used cannabis since their diagnosis, with 20.1% reporting current use and 23.9% use during cancer treatment (Table 2). Rural patients reported slightly higher cannabis use rates than urban/suburban patients, although these differences were not statistically significant. In multivariable analysis (data not shown), area of residence (rural vs. urban/suburban) was not significantly associated with cannabis use since diagnosis (*p* = 0.801), with age the only significant predictor (older age predicting lower use, *p* < 0.01).

**Table 2:** Cannabis use and key attitudes, stratified by area of residence

	Overall (*n* = 186)		Area of Residence (*n *= 179)		
			Rural (*n* = 92)	Urban/Suburban (*n* = 87)	
	Response (*n*)	% (95% CI)	%	%	*p*
Used since diagnosis	184	26.1 (20.0–33.2)	30.0	23.0	0.31
Currently using	184	20.1 (14.7–26.8)	23.9	16.3	0.26
Used during cancer treatment	182	23.9 (18.0–30.9)	28.6	19.3	0.16
Used as anti-cancer treatment	183	9.8 (6.1–15.3)	8.9	11.6	0.62
Used since cancer treatment†	59	41.2 (27.9–55.8)	46.4	34.8	0.57
Used instead of opioids to manage pain†	73	30.1 (20.2–42.2)	36.8	24.2	0.31
Openness to use	183				0.45
- *Mostly/very open*		55.8 (48.2–63.0)	58.9	52.9	
- *Somewhat open/not open*		44.2 (37.0–51.8)	41.1	47.1	
Thinks there are benefits to using	184	92.9 (88.0–96.0)	92.4	92.9	1.0
Thinks there are risks to using	180	76.7 (69.7–82.5)	70.0	82.1	0.08

Response (*n*) indicates the number of respondents with non-missing data for each survey item; percentages and 95% CIs were calculated among respondents to that item. Area-specific percentages and p values are based on respondents who also reported area of residence

†Based on questionnaire instructions, these items were to be completed only by respondents with lifetime cannabis use; the displayed Response (*n*) values are item-specific denominators

The most common mode of cannabis use was smoking (52.2% of users). When asked what the primary components of their cannabis products were, delta-9 THC was the most commonly reported (45.6% of users), followed by CBD (26.1%), and Delta-8 THC (19.6%), with 28.3% being unsure. 71.4% of users were obtaining it from state-regulated dispensaries, with urban/suburban patients more likely than rural patients to do so (*p* = 0.046). 44.9% of users consumed cannabis almost every day or multiple times a day, while 32.7% consumed it once a month or less. Among respondents answering follow-up items that were to be completed only by those who had ever used cannabis, 30.1% reported using cannabis instead of opioids to manage pain, and 32.0% reported that cannabis helped them decrease or stop other medications.

**Fig. 1:**
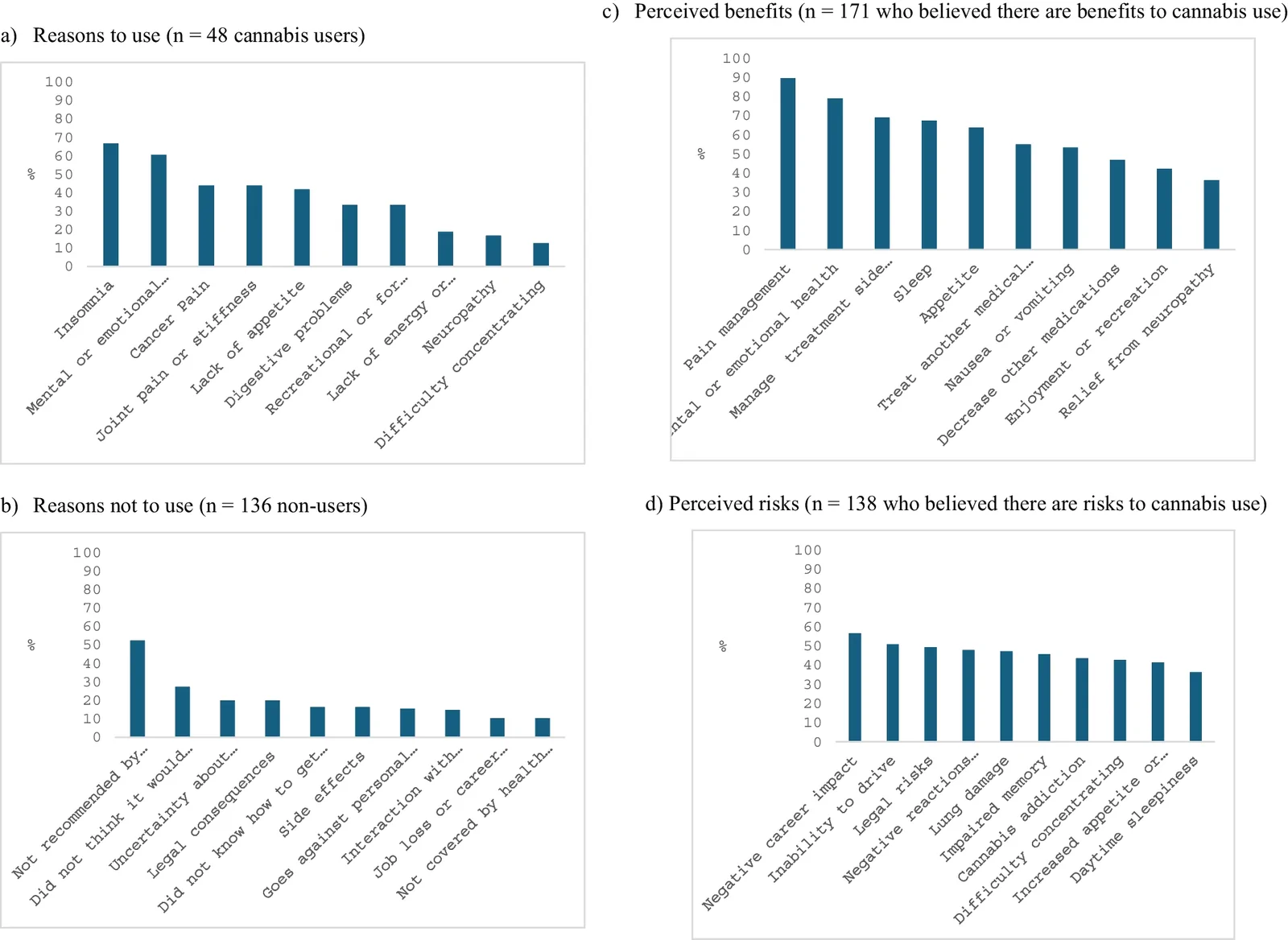
Most common reasons to use or not use cannabis and perceived benefits and risks to use. **a** Reasons to use (*n* = 48 cannabis users). **b** Reasons not to use (*n* = 136 non-users). **c** Perceived benefits (*n* = 171 who believed there are benefits to cannabis use). **d** Perceived risks (*n* = 138 who believed there are risks to cannabis use)

Respondents who used cannabis after diagnosis were asked separately about their reasons for use, including symptom-related reasons and recreational use/enjoyment. The most common reasons were sleep aid (66.7% of users), mood/stress/anxiety/depression (60.4%), cancer pain (43.8%), and joint pain/stiffness (43.8%) (Fig. 1a). The most common reasons for not using cannabis were that their healthcare providers had not recommended or suggested it (52.2% of non-users), patients believing it would not be helpful (27.2%), being concerned about legal consequences (19.9%), or believing there were too many choices/being unsure about which products were safe and effective (19.9%) (Fig. 1b). Compared to rural patients, urban/suburban patients were more likely to avoid using cannabis due to concerns about its interaction with other medications (23.9% vs. 4.8%, *p* = 0.002).

**Fig. 2:**
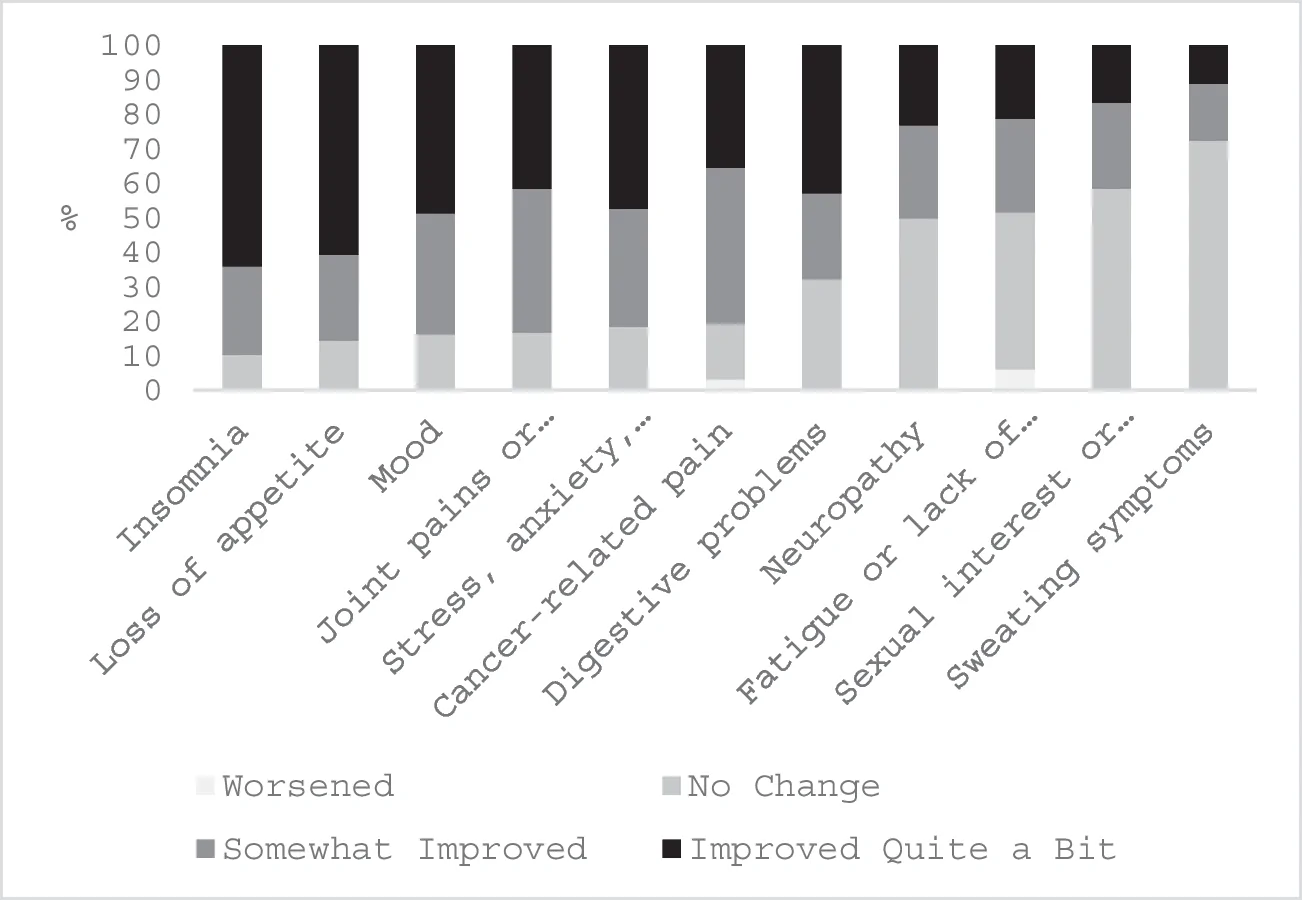
Self-reported changes in cancer-related symptoms due to cannabis use, in order of most to least improved symptoms (*n* = 46 cannabis users)

Most cannabis users reported that it improved their cancer-related symptoms for 7 out of 11 assessed symptoms (Fig. 2). The most improved symptoms were insomnia (89.7%), appetite (85.7%), mood (83.8%), joint pain/stiffness (83.3%), stress/anxiety/depression (81.6%), cancer-related pain (80.7%), and digestive symptoms (67.9%). The symptoms reported to be least improved were sweating/hot flashes (27.8%), sexual interest (41.7%) and fatigue/lack of energy (48.5%), which was the symptom most reported to have worsened due to cannabis (6.1%).

83.1% of patients were at least somewhat open to using cannabis, with 55.7% mostly or very open. 55.5% thought that cannabis is safe for cancer patients to use, with only 2.8% believing it unsafe. 92.9%, including those who had not used cannabis, thought there are benefits related to its use, primarily pain management (89.5%), relief of stress/anxiety/depression (79.0%), and managing side effects from cancer treatment (69.0%) (Fig. 1c).

Urban/suburban patients were more likely to believe that cannabis helps with nausea or vomiting (63.3% vs. 44.7%, *p* = 0.019). However, 76.7% of patients also thought there are risks related to cannabis use, mostly in the legal or social realm such as negative career effects (56.5%), being unable to drive (50.7%), facing legal risks (49.3%), and experiencing negative reactions from family members or friends (47.8%) (Fig. 1d). Rural patients were more likely to believe that there is an increased risk of cancer due to cannabis use than urban/suburban patients (17.5% vs. 5.8%, *p* = 0.053). A similar proportion of respondents believed that cannabis is addictive (33.6%) compared to those who viewed it as non-addictive (34.6%).

When asked if they would be willing to participate in research with cannabis, 49.2% reported being likely or very likely to consider doing so, with a similar level of interest between rural and urban/suburban patients. The cannabis preparations most likely to be of interest were in the form of food, followed by topical applications, oral intake, and in liquid form. The THC content in cannabis was a strong consideration for research participation, with 21.6% reporting that they would be more likely to participate in research with THC-containing cannabis compared to 7.8% who would be less likely to participate. Finally, patients were open to obtaining the cannabis from a dispensary if directed to do so, with 66.7% in favor compared to 13.7% who were unwilling to do so.

## Discussion

In this anonymous survey of 186 cancer patients investigating cannabis use and attitudes in a state where patients have access to medical but not recreational cannabis, 26% reported having used cannabis since their cancer diagnosis, while 20% reported current use. Most users reported improvement in 7 of 11 cancer-related symptoms. 93% perceived benefits to its use and 83% were open to use, though 77% also acknowledged risks. The proportion who had used cannabis since their diagnosis is slightly higher than recent estimates from national samples of cancer patients and survivors, though it falls within the range reported by single-site studies. The relatively high rate may reflect sample composition, including the large proportion of breast cancer patients (28%), of whom 36% reported post-diagnosis use; a recent survey of U.S. breast cancer patients likewise reported 42% cannabis use for symptom relief [37].

Cannabis users reported that it was effective in managing a range of symptoms, aligning with findings from other studies [13, 23], and suggesting a potential role in symptom self-management. Respondents most often reported improved insomnia, appetite, and mood, broadly aligning with prior reports from Raghunathan et al. on appetite and sleep [24] and Azizoddin et al. on sleep, nausea/vomiting, headaches, and pain. [18] However, reflecting limited high-quality clinical evidence, ASCO’s 2024 guideline made only a weak recommendation to consider cannabis-based medicines as adjuncts for refractory CINV, with insufficient evidence to recommend their use for other cancer-related symptoms or treatment-related toxicities [25]. Notably, GI symptoms including nausea/vomiting ranked 6th among reasons for use in our study, below insomnia, appetite, pain, and mental/emotional health. Importantly, most randomized trials in this research area have evaluated standardized cannabis-based medicines (e.g., dronabinol, nabilone, nabiximols) rather than the inhaled/whole-plant products many patients use, so generalization from these trials to real-world use should be cautious. High-quality, regimen-concordant trials are needed to clarify net benefit–risk and which products, doses, and indications are most beneficial (if any) in routine care.

Our sample generally viewed cannabis as safe and beneficial, with 83% at least somewhat open to using it, 93% believing there are benefits to its use, and only 3% considering it unsafe, consistent with prior work [18, 23]. However, 77% of participants expressed concern about its risks, especially legal, professional, and social consequences. The lack of healthcare provider endorsement as well as the barriers and risks associated with its use reflect ongoing challenges in integrating cannabis into clinical practice. Given the lack of quality evidence on its benefits in cancer populations, the potential for legal liability, a lack of relevant education in this field and the limited federal oversight of cannabis production, oncologists report significant uncertainty about providing guidance regarding cannabis to patients, who in turn often seek nonmedical sources for advice [25].

In our data, cannabis use did not differ significantly by rural versus urban/suburban residence, and age was the only significant predictor of use since diagnosis in the multivariable model. This aligns with a national BRFSS analysis among cancer survivors [1] and two cancer-center cohorts reporting no rural–urban differences [18, 38], although some general-population studies have found higher cannabis rates in urban areas [8, 39]. Brasky et al. also found higher cannabis prevalence, more intensive use, and lower medical-cannabis program participation in more socioeconomically deprived areas, raising the possibility that area-level deprivation may be more relevant than rurality per se. [38] However, this interpretation remains hypothesis-generating in our study: we lacked ZIP code–linked deprivation measures, individual income and education were not significantly associated with cannabis use, and our modest sample limited detection of associations other than strong predictors such as age. Descriptively, rural respondents were less likely than urban/suburban respondents to report living comfortably on their present income and were somewhat more likely to report cannabis use since diagnosis (30% vs. 23%), although neither difference reached statistical significance. Future studies linking patient ZIP codes to deprivation indices may help distinguish individual socioeconomic factors from area-level barriers, including transportation, dispensary access, certification costs, and availability of symptom-management education.

Rural and urban/suburban patients also reported similar attitudes towards cannabis. Although a recent study from Pennsylvania found that adults from urban areas were more likely than those from rural areas to disclose their cannabis use to healthcare providers [40], potentially suggesting a greater stigma around cannabis use among those from rural areas, our study did not find differences between these groups on questions about stigma or comfort in discussing cannabis with healthcare providers. However, additional differences between these groups in cannabis use and attitudes might have reached statistical significance with a larger sample size. Still, the lower use of dispensaries among rural users, lower concern about medication interactions among rural non-users, and borderline greater concern about cancer risk among rural respondents may point to different cannabis-related educational needs by residence. These findings support accessible, clinician-led counseling about cannabis products, sourcing, expected benefits and risks, and potential drug–drug interactions, particularly for oncology practices serving mixed rural–urban catchments.

The study’s findings have several implications for clinical practice and policy. The openness to cannabis use expressed by a large majority of the sample (83%) and the interest in participating in cannabis-related research (49%) are encouraging, given the need for more clinical trials to develop evidence-based guidelines, as recommended by ASCO [25]. Recent federal rescheduling of certain FDA-approved cannabis-containing drug products and state-licensed medical cannabis products to Schedule III may reduce some research barriers, although the practical impact for oncology studies remains uncertain [6]. Almost half (45%) of the patients reported a desire to receive information from their physicians about cannabis use, pointing to a potential gap in patient-provider communication around the risks and benefits of use for people with cancer. Across recent reviews and surveys, cannabis conversations in oncology are often patient-initiated and inconsistent, with substantial provider knowledge gaps and lower comfort or disclosure among younger or newly diagnosed patients and in non-legal settings [41–43]. Evidence suggests that brief curricula can boost trainee confidence [44], and destigmatizing conversations that address safety and potential drug–drug interactions are recommended [45].

Another practical implication of our study is our finding that our sample had commonly used cannabis as an alternative to opioids for pain management. Nearly one-third of users (30%) reported substituting cannabis for opioids, and 32% reported reducing or stopping other medications. This supports existing evidence suggesting that many patients prefer cannabis over opioids due to fewer side effects and better symptom control [14, 46], and is consistent with evidence that many cancer patients report reducing their use of prescription opioids after initiating cannabis for symptom management [31]. Despite patient-reported relief and strong demand, randomized trials and meta-analyses have failed to show clinically meaningful analgesia from cannabinoids for cancer pain, with high-quality evidence indicating at best modest, inconsistent effects alongside higher adverse events [47–49]. Further work should identify which patients might benefit from cannabis use, and test formulations, dosing, and routes, including whole-plant/inhaled products, using rigorous designs that can adjudicate analgesic efficacy and opioid-sparing effects.

Our study had several strengths. By assessing differences in cannabis use and attitudes among rural and urban/suburban cancer patients, we addressed a gap in knowledge. The in-person survey approach helped include rural patients who may be underrepresented in online surveys because of limited internet access or computer literacy. The detailed questionnaire also captured real-world usage patterns and patient attitudes, complementing RCTs that have typically evaluated cannabis-based medicines rather than whole-plant products.

Limitations include the cross-sectional design, which precludes causal inference, and reliance on self-reported measures, which may be subject to recall and social desirability biases. Urbanicity was based on self-reported residence rather than addresses or ZIP codes, preventing more granular analyses using measures such as the Social Deprivation Index [38]. Given the exploratory nature of our analyses and the large number of rural vs. urban/suburban comparisons, we did not adjust for multiple comparisons, increasing the risk of type I error. We also did not differentiate product characteristics such as THC/CBD ratios, dose, formulation, or route, which may influence effects [23]. The single-institution setting, modest sample size, and racially non-diverse sample limit generalizability. Finally, voluntary participation precluded assessment of non-responders, and recruitment during in-person oncology visits likely oversampled patients on active treatment, patients with later-stage disease, and breast cancer patients, who were younger than patients with other cancers in our sample (data not shown).

In conclusion, cannabis use was relatively common among our sample of cancer patients, who held generally favorable attitudes towards its use, with most cannabis users reporting symptom relief across a majority of cancer-related symptoms assessed. Broadly similar cannabis attitudes and rates of use were observed between rural and urban/suburban patients. These findings point to the potential therapeutic benefits of cannabis among cancer populations. Future work should include clinical trials and multi-site prospective studies across diverse regions that assess safety and effectiveness, explicitly evaluate quality-controlled whole-plant and inhaled formulations, and test clinically meaningful endpoints, such as opioid-sparing effects and treatment interactions.

## Data Availability

The data analyzed in this study are available to be shared on request to the corresponding author.

## References

[1] Lee M, Salloum RG, Jenkins W, Hales DB, Sharma A (2023) Marijuana use among US adults with cancer: findings from the 2018-2019 Behavioral Risk Factor Surveillance System. J Cancer Surviv 17(4):1161–1170. 10.1007/s11764-021-01138-z 35397761

[2] Salz T, Meza AM, Chino F, Mao JJ, Raghunathan NJ, Jinna S, Brens J, Furberg H, Korenstein D (2023) Cannabis use among recently treated cancer patients: perceptions and experiences. Support Care Cancer 31(9):545. 10.1007/s00520-023-07994-y PMC10585595 37650961

[3] Ellison GL, Helzlsouer KJ, Rosenfield SM, Kim Y, Ashare RL, Blaes AH, Cullen J, Doran N, Ebbert JO, Egan KM, Heffner JL, Lee RT, McClure EA, McDaniels-Davidson C, Meghani SH, Newcomb PA, Nugent S, Hernandez-Ortega N, Salz T, Vidot DC, Worster B, Zylla DM (2024) Perceptions, prevalence, and patterns of cannabis use among cancer patients treated at 12 NCI-Designated Cancer Centers. J Natl Cancer Inst Monogr 2024(66):202–217. 10.1093/jncimonographs/lgae029 PMC11303851 39108244

[4] Ton M, Newcomb PA, Jones S, Malen RC, Heffner JL (2024) Cannabis use after a cancer diagnosis in a population-based sample of cancer survivors. Cancer Causes Control 35(7):1033–1042. 10.1007/s10552-024-01860-w PMC12893207 38519643

[5] National Conference of State Legislatures. State medical cannabis laws. Updated January 21, 2025. https://www.ncsl.org/health/state-medical-cannabis-laws. Accessed 5 Nov 2025

[6] Drug Enforcement Administration, Department of Justice (2026) Schedules of controlled substances: Rescheduling of food and drug administration approved products containing marijuana from schedule i to schedule III; Corresponding change to permit requirements. Fed Reg 91(81):22714–22723

[7] Cousins MM, Mayo C, Devasia T, Dykstra M, Regan S, Miller S, Allen SG, Bryant AK, Morales-Rivera K, Herr DJ, Edwards DM, Takayesu J, Birer S, Egerer N, Evans J, Elliott D, Henderson C, Laucis AM, McFarlane M, Dragovic AF, Shah J, Hayman JA, Coughlin LN, Ilgen M, Jagsi R (2023) Cannabis use in patients seen in an academic radiation oncology department. Pract Radiat Oncol 13(2):112–121. 10.1016/j.prro.2022.11.002 PMC9996408 36460181

[8] Coughlin LN, Bonar EE, Bohnert KM, Jannausch M, Walton MA, Blow FC, Ilgen MA (2019) Changes in urban and rural cigarette smoking and cannabis use from 2007 to 2017 in adults in the United States. Drug Alcohol Depend 205:107699. 10.1016/j.drugalcdep.2019.107699 PMC6951810 31707265

[9] Substance Abuse and Mental Health Services Administration (2021) Key substance use and mental health indicators in the United States: Results from the 2020 National survey on drug use and health. HHS Publication No. PEP21–07–01–003, DSDUH Series H-56. Center for behavioral health statistics and quality, substance abuse and mental health services administration. https://www.samhsa.gov/data

[10] Tringale KR, Huynh-Le MP, Salans M, Marshall DC, Shi Y, Hattangadi-Gluth JA (2019) The role of cancer in marijuana and prescription opioid use in the United States: a population-based analysis from 2005 to 2014. Cancer 125(13):2242–2251. 10.1002/cncr.32059 PMC6810711 31006849

[11] Azagba S, Shan L, Manzione L (2020) Cigarette, e-cigarette, alcohol, and marijuana use by cancer diagnosis status: a longitudinal analysis. Subst Abuse 14:1178221820980470. 10.1177/1178221820980470 PMC7756047 33424226

[12] Do EK, Ksinan AJ, Kim SJ, Del Fabbro EG, Fuemmeler BF (2021) Cannabis use among cancer survivors in the United States: analysis of a nationally representative sample. Cancer 127(21):4040–4049. 10.1002/cncr.33794 PMC8600683 34387864

[13] Amin S, Chae SW, Kawamoto CT, Phillips KT, Pokhrel P (2024) Cannabis use among cancer patients and survivors in the United States: a systematic review. JNCI Cancer Spectr 8(1):pkae004. 10.1093/jncics/pkae004 PMC10868394 38291891

[14] Reblin M, Sahebjam S, Peeri NC, Martinez YC, Thompson Z, Egan KM (2019) Medical cannabis use in glioma patients treated at a comprehensive cancer center in Florida. J Palliat Med 22(10):1202–1207. 10.1089/jpm.2018.0528 PMC7364313 31081711

[15] Meghani SH, Quinn R, Ashare R, Levoy K, Worster B, Naylor M, Chittams J, Cheatle M (2021) Impact of cannabis use on least pain scores among African American and White patients with cancer pain: a moderation analysis. J Pain Res 14:3493–3502. 10.2147/JPR.S332447 PMC8577564 34764689

[16] Baral A, Diggs BA, Marrakchi El Fellah R, McCarley C, Penedo F, Martinez C, Vidot DC (2024) Cannabis use among cancer patients during active treatment: findings from a study at an NCI-designated cancer center. Cancer Med 13(21):e70384. 10.1002/cam4.70384 PMC11530714 39487679

[17] Donovan KA, Portman DG (2021) Effect of COVID-19 Pandemic on Cannabis Use in Cancer Patients. Am J Hosp Palliat Care 38(7):850–853. 10.1177/1049909121999784 33657874

[18] Azizoddin DR, Cohn AM, Ulahannan SV, Henson CE, Alexander AC, Moore KN, Holman LL, Boozary LK, Sifat MS, Kendzor DE (2023) Cannabis use among adults undergoing cancer treatment. Cancer 129(21):3498–3508. 10.1002/cncr.34922 PMC11070130 37354093

[19] Adley NC, Brasky TM, Conroy SR, Newton AM, Plascak JJ, Strassels SA, Hays JL, Krok-Schoen JL (2024) Associations of cancer patients’ demographic and clinical characteristics with cannabis-related interest and behaviors. J Palliat Med 27(3):394–399. 10.1089/jpm.2023.0490 PMC11447484 38157334

[20] Poghosyan H, Poghosyan A (2021) Marijuana use among cancer survivors: quantifying prevalence and identifying predictors. Addict Behav 112:106634. 10.1016/j.addbeh.2020.106634 32920457

[21] Osaghae I, Talluri R, Chido-Amajuoyi OG, Tanco K, Shah DP, Pande M, Shete S (2024) Awareness and interest in cannabis use for cancer management among cancer survivors. Cancer Med 13(1):e6902. 10.1002/cam4.6902 PMC10807618 38180296

[22] Islam JY, Nguyen OT, Turner K, Martinez YC, Rodriguez OG, Rodriguez DI, Rajasekhara S, Chang YD, Gonzalez BD, Jim HSL, Egan KM (2024) Concurrent substance use among cancer patients with and without a history of cannabis use since cancer diagnosis at an NCI-Designated Cancer Center in Florida. J Natl Cancer Inst Monogr 2024(66):224–233. 10.1093/jncimonographs/lgad036 PMC11303859 39108241

[23] Brasky TM, Newton AM, Conroy S, Adib A, Adley NC, Strassels SA, Hays JL, Cooper ZD, Wagener TL, Stevens E, Plascak JJ, Krok-Schoen JL (2023) Marijuana and Cannabidiol Use Prevalence and Symptom Management Among Patients with Cancer. Cancer Res Commun 3(9):1917–1926. 10.1158/2767-9764.CRC-23-0233 PMC10515742 37772996

[24] Raghunathan NJ, Brens J, Vemuri S, Li QS, Mao JJ, Korenstein D (2022) In the weeds: a retrospective study of patient interest in and experience with cannabis at a cancer center. Support Care Cancer 30(9):7491–7497. 10.1007/s00520-022-07170-8 PMC9165925 35665859

[25] Braun IM, Bohlke K, Abrams DI, Anderson H, Balneaves LG, Bar-Sela G, Bowles DW, Chai PR, Damani A, Gupta A, Hallmeyer S, Subbiah IM, Twelves C, Wallace MS, Roeland EJ (2024) Cannabis and cannabinoids in adults with cancer: ASCO guideline. J Clin Oncol 42(13):1575–1593. 10.1200/JCO.23.02596 PMC11730458 38478773

[26] Creangă-Murariu I, Rezuș II, Karami R, Rancz A, Zolcsák Á, Engh MA, Obeidat M, Tamba BI, Hegyi P, Bunduc S (2025) Indications of cannabinoids for the palliation of cancer-associated symptoms: a systematic review and meta-analysis. Curr Oncol Rep 27(9):1080–1096. 10.1007/s11912-025-01695-x PMC12450232 40748522

[27] Solmi M, De Toffol M, Kim JY, Choi MJ, Stubbs B, Thompson T, Firth J, Miola A, Croatto G, Baggio F, Michelon S, Ballan L, Gerdle B, Monaco F, Simonato P, Scocco P, Ricca V, Castellini G, Fornaro M, Murru A, Vieta E, Fusar-Poli P, Barbui C, Ioannidis JPA, Carvalho AF, Radua J, Correll CU, Cortese S, Murray RM, Castle D, Shin JI, Dragioti E (2023) Balancing risks and benefits of cannabis use: umbrella review of meta-analyses of randomised controlled trials and observational studies. BMJ 382:e072348. 10.1136/bmj-2022-072348 PMC10466434 37648266

[28] Camilleri M, Zheng T (2023) Cannabinoids and the gastrointestinal tract. Clin Gastroenterol Hepatol 21(13):3217–3229. 10.1016/j.cgh.2023.07.031 PMC10872845 37678488

[29] Poghosyan H, Noonan EJ, Badri P, Braun I, Young GJ (2021) Association between daily and non-daily cannabis use and depression among United States adult cancer survivors. Nurs Outlook 69(4):672–685. 10.1016/j.outlook.2021.01.012 33581859

[30] Kim Y, Huh J, Miller KA, Ritt-Olson A, Hoyt MA, Milam J (2024) Clinical, demographic factors, and substance use among Hispanic and non-Hispanic young adult childhood cancer survivors. J Psychosoc Oncol 42(6):793–810. 10.1080/07347332.2024.2326148 PMC11415543 38513227

[31] Blake EA, Ross M, Ihenacho U, Figueroa L, Silverstein E, Flink D, Mendez-Ishizaki Y, Yessaian A, Brunette LL, Matsuo K, Cortessis VK, Guntupalli S, Roman L (2019) Non-prescription cannabis use for symptom management amongst women with gynecologic malignancies. Gynecol Oncol Rep 30:100497. 10.1016/j.gore.2019.100497 PMC6804884 31692541

[32] Levit LA, Byatt L, Lyss AP, Paskett ED, Levit K, Kirkwood K, Schenkel C, Schilsky RL (2020) Closing the rural cancer care gap: three institutional approaches. JCO Oncol Pract 16(7):422–430. 10.1200/OP.20.00174 32574128

[33] Henley SJ, Anderson RN, Thomas CC, Massetti GM, Peaker B, Richardson LC (2017) Invasive cancer incidence, 2004-2013, and deaths, 2006-2015, in nonmetropolitan and metropolitan counties - United States. MMWR Surveill Summ 66(14):1–13. 10.15585/mmwr.ss6614a1 PMC5879727 28683054

[34] Marks BJ, Liao J, Lam C, Moeckel C, Lengerich EJ (2025) Harvesting risk: an ecologic study of agricultural practices and patterns and melanoma incidence in Pennsylvania. JCO Clin Cancer Inform 9:e2500160. 10.1200/CCI-25-00160 PMC12629121 41237350

[35] Epidemiology and Genomics Research Program NCI. Cannabis core measures questionnaire. https://epi.grants.cancer.gov/cannabis/CoreMeasuresQuestionnaire.pdf. Accessed 6 Nov 2025

[36] R Core Team (2024) R: A language and environment for statistical computing. R foundation for statistical computing, Vienna, Austria. https://www.R-project.org

[37] Weiss MC, Hibbs JE, Buckley ME, Danese SR, Leitenberger A, Bollmann-Jenkins M, Meske SW, Aliano-Ruiz KE, McHugh TW, Larson SL, Le EH, Green NL, Gilman PB, Kaklamani VG, Chlebowski RT, Martinez DM (2022) A Coala-T-Cannabis survey study of breast cancer patients’ use of cannabis before, during, and after treatment. Cancer 128(1):160–168. 10.1002/cncr.33906 PMC9413357 34636036

[38] Brasky TM, Lee S, McBride B, Newton AM, Baltic RD, Wagener TL, Conroy S, Hays JL, Stevens EE, Adib A, Krok-Schoen JL (2025) Prevalence of cannabis and medication use by indices of residential urbanicity and deprivation among Ohio cancer patients. Cancer Causes Control 36(7):719–724. 10.1007/s10552-025-01972-x PMC12103373 39934610

[39] Coughlin LN, Walton MA, McCormick R, Blow FC (2020) Prevalence and severity of alcohol and cannabis use across the urban-rural continuum in the Michigan National Guard. J Rural Health 36(2):234–239. 10.1111/jrh.12412 PMC7102927 31840327

[40] Mallinson DJ, Servinsky TJ Jr. (2024) Rural reticence to inform physicians of cannabis use. J Rural Health. 10.1111/jrh.12885 PMC11950414 39320049

[41] Baral A, Diggs BA, Aka A, Williams R, Ortega NH, Fellah RME, Islam JY, Camacho-Rivera M, Penedo FJ, Vidot DC (2025) Experiences and comfort of young cancer patients discussing cannabis with their providers: insights from a survey at an NCI-designated cancer center. J Cancer Educ 40(2):256–265. 10.1007/s13187-024-02507-9 PMC11978675 39294413

[42] Baral A, Diggs BA, Greengold J, Foronda C, Anglade D, Camacho-Rivera M, Islam JY, Vidot DC (2025) State of the science: health care provider communication of cannabis use among adults living with cancer. J Cancer Educ 40(3):329–347. 10.1007/s13187-024-02484-z PMC12177019 39160419

[43] Wedel AV, Walters KJ, Tomko RL, Rojewski AM, McClure EA (2025) Evaluating racial disparities in cancer patient-provider communication about cannabis in a state without a legal cannabis marketplace. Support Care Cancer 33(2):78. 10.1007/s00520-024-09131-9 PMC11711712 39775254

[44] Patell R, Bindal P, Freed J, Dodge LE, Nagaraj G, LaCasce AS, Elkon J, Im A, Suzuki S, Yedavalli N, Winegarden JD, Buss MK, Schonberg MA, Braun IM, Rangachari D (2025) A multicenter feasibility study of a novel curriculum for oncology trainees regarding medical cannabis. J Natl Compr Canc Netw 23(3):82–89. 10.6004/jnccn.2024.7084 PMC12934205 40073833

[45] Worster B, Hajjar ER, Handley N (2022) Cannabis use in patients with cancer: a clinical review. JCO Oncol Pract 18(11):743–749. 10.1200/OP.22.00080 35749680

[46] Krok-Schoen JL, Plascak JJ, Newton AM, Strassels SA, Adib A, Adley NC, Hays JL, Wagener TL, Stevens EE, Brasky TM (2024) Current cannabis use and pain management among US cancer patients. Support Care Cancer 32(2):111. 10.1007/s00520-024-08321-9 PMC10796435 38236449

[47] Wang L, Hong PJ, May C, Rehman Y, Oparin Y, Hong CJ, Hong BY, AminiLari M, Gallo L, Kaushal A, Craigie S, Couban RJ, Kum E, Shanthanna H, Price I, Upadhye S, Ware MA, Campbell F, Buchbinder R, Agoritsas T, Busse JW (2021) Medical cannabis or cannabinoids for chronic non-cancer and cancer related pain: a systematic review and meta-analysis of randomised clinical trials. BMJ 374:n1034. 10.1136/bmj.n1034 34497047

[48] Häuser W, Welsch P, Radbruch L, Fisher E, Bell RF, Moore RA (2023) Cannabis-based medicines and medical cannabis for adults with cancer pain. Cochrane Database Syst Rev 6(6):CD014915. 10.1002/14651858.CD014915.pub2 PMC10241005 37283486

[49] To J, Davis M, Sbrana A, Alderman B, Hui D, Mukhopadhyay S, Bouleuc C, Case AA, Amano K, Crawford GB, de Feo G, Tanco K, Garsed J (2023) MASCC guideline: cannabis for cancer-related pain and risk of harms and adverse events. Support Care Cancer 31(4):202. 10.1007/s00520-023-07662-1 36872397

